# Conveying practical clinical skills with the help of teaching associates—a randomised trial with focus on the long term learning retention

**DOI:** 10.1186/s12909-017-0892-5

**Published:** 2017-03-28

**Authors:** Sebastian H. Hoefer, Jasmina Sterz, Bernd Bender, Maria-Christina Stefanescu, Marius Theis, Felix Walcher, Robert Sader, Miriam Ruesseler

**Affiliations:** 1 Department of Oral, Cranio-Maxillofacial, and Facial Plastic Surgery, University Hospital Frankfurt, Goethe University, Theodor-Stern-Kai 7, 60590 Frankfurt, Germany; 2 Department of Trauma, Hand, and Reconstructive Surgery, University Hospital Frankfurt, Goethe University, Theodor-Stern-Kai 7, 60590 Frankfurt, Germany; 3 Department of Trauma Surgery, Medical Faculty University Hospital Magdeburg, Leipziger Str. 44, 39120 Magdeburg, Germany

**Keywords:** Medical education, Teaching associates, Complex practical skills, Long term benefit, Long term evaluation, Multimodal feedback

## Abstract

**Background:**

Ensuring that all medical students achieve adequate clinical skills remains a challenge, yet the correct performance of clinical skills is critical for all fields of medicine. This study analyzes the influence of receiving feedback by teaching associates in the context of achieving and maintaining a level of expertise in complex head and skull examination.

**Methods:**

All third year students at a German university who completed the obligatory surgical skills lab training and surgical clerkship participated in this study. The students were randomized into two groups. Control group: lessons by an instructor and peer-based practical skills training. Intervention group: training by teaching associates who are examined as simulation patients and provided direct feedback on student performance. Their competency in short- and long-term competence (directly after intervention and at 4 months after the training) of head and skull examination was measured.

Statistical analyses were performed using SPSS Statistics version 19 (IBM, Armonk, USA). Parametric and non-parametric test methods were applied. As a measurement of correlation, Pearson correlations and correlations via Kendall’s-Tau-b were calculated and Cohen’s d effect size was calculated.

**Results:**

A total of 181 students were included (90 intervention, 91 control). Out of those 181 students 81 agreed to be videotaped (32 in the control group and 49 in the TA group) and examined at time point 1. At both time points, the intervention group performed the examination significantly better (time point 1, *p* = <.001; time point 2 (rater 1 *p* = .009, rater 2 *p* = .015), than the control group. The effect size (Cohens d) was up to 1.422.

**Conclusions:**

The use of teaching associates for teaching complex practical skills is effective for short- and long-term retention. We anticipate the method could be easily translated to nearly every patient-based clinical skill, particularly with regards to a competence-based education of future doctors.

**Electronic supplementary material:**

The online version of this article (doi:10.1186/s12909-017-0892-5) contains supplementary material, which is available to authorized users.

## Background

Practical skills play a central role in daily clinical practice, [1]. Due to the complexity of the skills required, especially with regard to technical-manual and psychosocial skills, practical clinical competence and thus competence-oriented training is of essential importance to university teaching. Furthermore, during undergraduate medical training, certain practical skills (e.g. injections, suturing, etc.) should be mastered to ensure a high level of security for both, students and patients.

Thus, many medical licensing boards and medical societies around the world have called for the strengthening of practical clinical skills in undergraduate medical training, as they are currently deemed insufficient [2–5]. The majority of final year medical students, not only in Germany but worldwide, rate their practical skills training as average or even poor [6–11]. According to Elsenhans [12], students feel like they have received poor guidance from their practicing medical colleagues. Surgeons receive the lowest ranking in the survey, where only 10% of all students reported a very good supervision experience and 20% rated their learning experience as very poor.

On a superficial level, the transfer of practical skills appears simple since it resembles a student-master-relationship in certain ways. However, if one visualizes the process of learning from classroom to application in daily clinical practice, it becomes apparent that the process is multidimensional, complex, and combines various level of competence. The seemingly “easily learned” manual skills transform into a complex assignment for the learner. Furthermore, most instructors do not receive adequate training and may have no special didactic qualifications. They primarily use those teaching methods they experienced themselves as students, and which they may not have critically reflected on [13–18]. For this reason, it can be assumed that the transfer of practical and clinical skills does not occur systematically and occurs within the context of a suboptimal didactic approach.

Even among trained instructors, there is a lack of consensus regarding teaching methods, including how to perform a complex physical examination such as a head and skull examination, and how best to transfer knowledge in this context. Several approaches are possible, including mastery learning or mental training [19–21], peer teaching, e-learning, or video-based learning. Further studies have shown that certain kinds of feedback can be used to facilitate the transfer of basic skills [22, 23]. But there is an absolute lack of studies focusing on the long term effect. Another possibility has been introduced by Barrows in 1964 [24], who was the first to include simulated patients in clinical education. He first used simulated patients for clinical teaching around neurological diseases and later applied the technique to various specialties [25].

Teaching Associates / Teaching Assistants (TA) mark a major development. The TAs use their own bodies to transfer knowledge about examination techniques [26]. This method is especially common in gynecology, urology, and proctology [26–28]. “The students receive immediate feedback on their skills and practice until they get it right.” [27]. This is, according to our understanding, an ideal mechanism for knowledge transfer that can be applied to other examination techniques. The conceptual framework of the method is closely aligned to the guidance hypothesis. Furthermore, TA’s “are trained to teach exams in a standardized manner and do not have an experience base, or bias, like physicians to adapt or modify the exam [27]. For all those reasons, we decided to teach a structured head and skull examination via TAs.

The aim of the study was to compare two teaching methods regarding skills transfer of a structured head and skull examination, and to determine which method has the greatest short- and long-term success in transferring complex practical clinical skills. The underlying hypothesis states that lessons with peer TAs produce the best results on a short- and long-term basis for successful transfer of competence of a head and skull examination.

## Methods

The study was approved by the ethical commission of the university hospital of Frankfurt (Johann-Wolfgang Goethe University) and it was stated, that no further approval was required. The study was conducted according to the Declaration of Helsinki.

### Setting, dates, and participants

All third year undergraduate medical students who were completing their obligatory surgical internship (including a skills lab week with various modules [29] that covered each surgical department as well as basic surgical skills) were invited to participate. The students were informed about the type and process of the study and gave their written consent for participation, which they could withdraw at any time, including the ability to voluntarily choose to have their performance video recorded. Students who agreed to participate in the study but did not wish to be videotaped participated in all aspects of the study except for the video recording (see intervention and assessment description below).

In addition, the instructors, TAs, and standardized patients also signed an informed consent form to participate in the study and provided permission to be videotaped in the examination.

### Instructors and teaching associates

The instructors and the TAs participated in a training session (240 min) prior to the start of the course. The training included exercises in regard to giving feedback in and performing the “head and skull examination”.

As instructors we appointed senior physicians from the Department of Cranio-Maxillofacial Surgery (CMF-Surgery) and as TAs we chose advanced students from a pool of students working for the department of surgery. The students were payed 10€/h.

### Randomization and intervention

A total of 181 students were randomized into two groups via balanced simple randomization. Balanced simple randomization aimed to reach nearly equal group sizes and to provide imbalance of gender between the groups. The control group consists of *n* = 91 students and the intervention group of *n* = 90 students. For both groups, the structured head and skull examination took place in the first part of the cranio-maxillofacial surgery module.

The module lasts 210 min and has a detailed time schedule (see Additional file 1) and a training manual, including a detailed description of all items included in the examination.

The control group received lessons by an instructor from the Department of CMF-Surgery. The sub-lecture “head and skull examination” included a structured power point presentation that covered examination techniques, a demonstration of the examination using a student, and practice performing the examination in a one-on-one peer-based context under the supervision and with feedback of the instructor.

The intervention group also received the theoretical lessons and the demonstration by an instructor of the Department of CMF-Surgery described above. However, there was no one-on-one peer exercise. To stay in the time limit of the course, two TAs were deployed to lead the examination exercise.

### Feedback

The intervention group received feedback in terms of guidance theory by the TAs. During the exercise period it was a concurrent visuohaptic multimodal feedback. Based on the definitions by Sigrist [30] we defined „concurrent visuohaptic multimodal feedback”as an augmented feedback, meaning a feedback that is provided by the TA during the exercise. Visuohaptic implies that the feedback-information transferred by speech is also strongly perceived in a visual and haptic manner by the student.

After the first assessment phase the students additionally got one terminal feedback by the standardized patients.

The control group got also feedback in the sense of guidance theory by the instructor. It was also a concurrent feedback, but the direct feedback of the examination subject was left out. Also the control group received feedback after the first assessment in the same way the intervention group did.

### Assessment

At the end of the module, the students who agreed to be videotaped participated in a formative videotaped assessment in the context of an OSCE station. The examination was performed on a standardized patient and was recorded. Afterwards the videos were shown to two examiners who were blinded with regards to the group assignment. They assessed the performance of the students with the standardized checklist (Additional file 2) used for head and skull examinations. The checklist is used in OSCE’s since 2007 and has been described previously [31]. The validation process of the checklist has been presented on the annual congress of the DGMKG (german society for cranio-maxillofacial surgeons) in 2009. The examiners were a second year resident (i.e., at the beginning of clinical training) and an attending doctor in the Department of CMF-Surgery. Both examiners rated the video material in an independent manner and assessed the students according to the OSCE checklist.

Four months after the skills lab week and the internship, the surgical OSCE took place as an obligatory final exam (summative).

Videotaping the entire exam was not possible since all students did not agree to being videotaped. For this reason, two examiners were at the head & skull examination of the OSCE station and rated the students. One examiner was an attending doctor in the Department of CMF-Surgery, the other examiner was an attending doctor in a related surgical discipline. These examiners were not members of the faculty and were also blinded with regards to group assignment. All raters participated in the mandatory examiner training at the faculty, which consists of a 30 min online tutorial and a 30 min simulation of a video rating.

Furthermore, we requested the time and the way of preparation the students prepared for the final OSCE exam referring to the head & skull examination, with a structured questionnaire.

### Statistical methods

Statistical analyses were performed using SPSS Statistics version 19 (IBM, Armonk, USA). If a Gaussian distribution was not present in the data of the variable then non-parametric test methods were applied. If a Gaussian distribution was present then parametric test methods were applied. To test for significant mean differences, the averages of both groups were analyzed with the parametric *T*-test or with the non-parametric Kolmogorov-Smirnov-test and the non-parametric Mann-Whitney-*U*-test. The Cohen’s d effect size was calculated for the mean difference between both groups. As a measurement of correlation, Pearson correlations and correlations via Kendall’s-Tau-b were calculated.

## Results

### General results

A total of 181 students were included in this study; 60.8% were female (*n* = 110) and 39.2% were male (*n* = 71). This reflects the gender distribution of the class that semester.

Of the 181 students, 91 were assigned to the control group and 90 to the intervention group (TA group). The gender ratio of the control group was 54♀ : 37♂ and the gender ratio of the intervention group was 56♀ : 34♂. Of those 181 students, 81 agreed to be videotaped (32 in the control group (22♀ : 10♂) and 49 (29♀ : 20♂) in the TA group).

### Objective structured Clinical Examination - Checklist Part A: Practical Clinical Skills

**Fig. 1:**
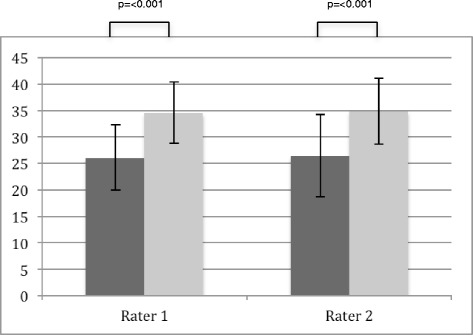
Group analysis point of time 1. Rater 1 attending physician CMF-surgery, Rater 2 resident physician CMF-surgery; *dark grey*—control group, *light grey*—intervention group; max score 48

**Fig. 2:**
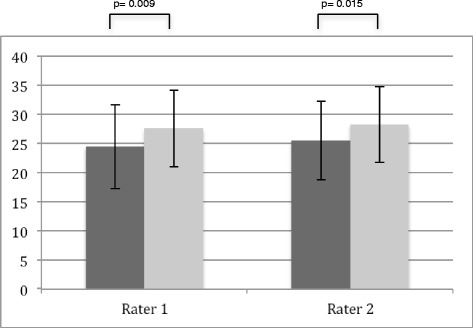
Group analysis point of time 2 (4 months after intervention). Rater 1 attending physician CMF-surgery, Rater 2 attending physician surgery; *dark grey*—control group, *light grey*—intervention group; max score 48

With regards to practical clinical skills, the TA group achieved significantly higher ratings from both raters at both points in time (time point 1, *p* = <.001; time point 2 (rater 1 *p* = .009, rater 2 *p* = .015), as compared to the control group (Figs. 1 and 2). At time point 1, female participants achieved slightly higher ratings than their male counterparts, however this difference was not significant (*p* = .173 and *p* = .201 for raters 1 and 2, respectively). However, at time point 2 the female students achieved significant higher results (*p* = <.001 and *p* = .015 for raters 1 and 2, respectively).

The effect size (Cohens d) for raters 1 and 2 at time point 1 was 1.422 and 1.201, whereas at time point 2 the effect size was .396 and .421, respectively.

### Objective structured Clinical Examination - Checklist Part B: Global Rating Scale

When comparing the items of the Global Rating Scale (GRS; assessed communication and interaction with patients), there were no significant differences between the two groups. However, the female students achieved significantly (rater 1 *p* = .034, rater 2 *p* = .002) better ratings than their male counterparts (Table 1).

**Table 1:** Analysis Part global rating scale (GRS) - communication & interaction skills

	Rater 1	Rater 2
♂	19.7	21.4
♀	+1.7	+1.4
*p* =	0.002	0.034

Multiple linear regression analysis; dependent variable: score; predictor: group; control variable: gender. The table shows the average sumscores of all 6 items on a 5 point likert scale (max. 30 points)

### Examiners

The inter-rater reliability was .895 (time point 1) and .944 (time point 2) respectively. Also analysis of the grading scores and the rating of the single items showed no significant differences between the examiners (*p* = .137).

### Duration of study

The structured questionnaire regarding the duration of self-study and the kind of preparation was answered by 94 students (48 control group, 46 TA group). The students in the control group used a median of 33.58 min (±24.14) to prepare for the examination. The students in the TA group used a median of 34.50 min (±29.66). A significant difference was not observed (*p* = .915). Analysis of study time with respect to gender also showed no significant differences (*p* = .867). If one correlates the length of study time with examination scores, a weak to slightly negative correlation can be found (−0.048 to −0.170). Furthermore, the type of studying behavior did not differ across students. With the exception of two students, most students stated that they studied with their fellow students.

## Discussion

Only a few studies analyze the use of TAs in practical clinical skills training for medical students. Furthermore, those studies predominantly deal with teaching the manual skills needed for examination of the urogenital and anal region, such as digital rectal examination or vaginal examination [32]. The Association of Standardized Patient Educators even introduced specialized nomenclature to describe these TAs (Gynecological Teaching Associates [GTA] and Male Urogenital Teaching Associates [MUTA]). Studies investigating the use of TAs in other contexts are rare. Barley et al. [27] described the use of TAs in multiple clinical disciplines. However, there is a gap in the literature regarding studies on the long-term efficacy of the use of TAs.

Our results from time point 1 (the significantly better results of the TA-group as well as the high effect size of the intervention) clearly demonstrate the superiority of the use of TAs in clinical examination instruction. We believe that the main reason for the success of the TA group is in the concurrent visuohaptic feedback on their examination techniques, which happens just as they are performing their examination. This assumption is supported by Sigrist and Hatala [30, 33]. Also the results by Hattie und Timperley [34] are in concordance with the findings of our study. They as well described effect sizes >1 in their metaanalysis of similarly natured feedback. The opportunity to get the haptic impression of the correct examination technique cannot be achieved in a peer exercise, even under supervision. Another reason could be the instructors themselves. TAs in our faculty are students who are at an advanced level of education. However, they are still “only” students. They are trained to follow the teaching manual and the teaching of examination techniques without divergence.

At time point 2, students taught by TAs remain significantly better than those in the control group. Even though at this point the measured effect size only values around 0.4 according to Hattie [34, 35] it is still within the”Zone of desired effects”. To our knowledge, ours is the first study to demonstrate that use of TAs to provide instruction on examination techniques delivers significantly better results, and that this difference persists at 4 months (Figs. 1 and 2). This long-term result is particularly telling, as it occurred during a summative final exam. Formative exams provide the students with constructive feedback according to their current level of knowledge and skills, whereas summative exams decide whether or not the students possess the qualifications necessary to complete a designated phase of the curriculum [36]. It is also known that exams—in accordance with the notion that “assessment drives learning”—are among the greatest motivators for students to deal with potential test material [37]. Henceforth, summative exams are more prone to bias they lead to more harmonized results. Despite this fact, at 4 months post-intervention we were still able to show a significant advantage with a useful effect size for TA-based instruction over usual practice. This proves the efficiency of this method, especially for achieving long-term results.

These results clearly support the research hypotheses that short- and long-learning success is higher when TAs are used for complex practical clinical skills.

Furthermore, female students at time point 2 achieved significantly better practical clinical skills scores than their male counterparts, in spite of no significant difference in length of time devoted to exam preparation. One possible explanation for this result lies within the assumption that female students prefer lessons that foster feedback, rational critique, and support. This assumption has been promoted by Schiefele, Mandl and Grüner [38, 39].

Our study has several limitations. First, participation voluntary, thus it is possible that only the motivated students took part in the videotaped formative OSCE evaluation. For this reason, we cannot determine if TAs had an effect on unmotivated students. However, this limitation is minimized by the data collected at time point 2, because all students who participated in the summative OSCE were rated.

Another limitation is the fact that the study was designed for a single center. Other universities might have different conditions that would prevent the findings from generalizing to their student populations.

Even though the study did not focus on communication skills with patients (i.e., assessed in the OSCE GRS), those results are worth mentioning. The female students achieved significantly better scores on the GRS than their male counterparts, regardless of group assignment. The cause of this difference cannot be explained by this study. However, it is possible that female students have a particular preference for skills involving social interaction (i.e., the skills assessed on the GRS). This interpretation could be supported by the fact that girls and women tend to score higher than men on assessments related to verbal competence [40]. Furthermore, interactive behavior and attitudes towards the learning topics is subjected to obvious gender differences [41]. To resolve this issue, future studies should concentrate on this issue.

Furthermore it has to be mentioned that a judgement if an interval of 4 months is long term or short term, is in the eye of the beholder and depends of the cut off. We defined for months as an long term interval.

Finally, also the economic side must be emblazed. Even in university hospitals the cost pressures in the health system can be felt and teaching and patient care are competing with each other. The ability to use teaching associates, proven to assure a high training standard provides us two advantages. In the local remuneration structure (two student TAs cost 10€/h each whereas the faculty member costs 50€/h), it is possible to save up to 60% of the costs by using TAs. Furthermore, it creates time for the medical faculty.

## Conclusion

The use of TAs to transfer the complex practical skills needed for a medical examination is an effective didactic method for both short- and long-term learning success. Due to the sustained impact in clinical skills, medical students like carry these improved skills into further aspects of their medical training. Furthermore, this method shows promise for easy translation to nearly every clinical skill that must be executed on the patient. In particular, it provides a promising platform for the competence-based education of future doctors.

## Additional files

Additional file 1: Blueprint of the CMF-surgery module. (DOCX 100 kb)

Additional file 2: Figure S1. OSCE Checklist CMFS (Part A) - practical clinical skills. **Figure S2.** OSCE Checklist CMFS (Part B) - Global Rating Scale (GRS). (DOCX 19 kb)

## Abbreviations

CMF-surgery: Cranio-maxillofacial surgey
DGMKG: Deutsche Gesellschaft für Mund-, Kiefer-, Gesichtschirurgie (german society for cranio-maxillofacial surgery)
GRS: Global rating scale
GTA: Gynecological Teaching Associate
MUTA: Male Urogenital Teaching Associate
OSCE: Objective structured clinical evaluation
TA: Teaching Associate
