# Supplementary material for: Conveying practical clinical skills with the help of teaching associates—a randomised trial with focus on the long term learning retention
Source: BMC Med Educ. 2017 Mar 28;17:65. doi: 10.1186/s12909-017-0892-5 (PMC5371235; doi:10.1186/s12909-017-0892-5)
Supplement: Supplementary file 1 — Blueprint of the CMF-surgery module. (DOCX 100 kb) [file 12909_2017_892_MOESM1_ESM.docx]

| Duration | Content | Methods | Protagonists |
| --- | --- | --- | --- |
| **15 min**  (15 min) | Welcome, Introduction,  Presentation of the learning goals | Frontal | Lecturer |
| **15 min**  (30 min) | Introduction in cranial examination | PPT | Lecturer |
| **60 min**  (90 min) | Practical exercise: cranial examination | Examination with TA or peer tandem | Lecturer/Teaching assistant and students |
| **5 min**  (95 min) | Break |  |  |
| **70 min**  (165 min) | Cranial traumatology | Case study:  Lower jaw fractures  Mid-face fractures  Fronto-basis fractures | Lecturer/students |
| **5 min**  (170 min) | Break |  |  |
| **30 min**  (200 min) | Cranio-maxillofacial emergencies | Buzz-groups | Lecturer/students |
| **10 min**  (210 min) | Conclusion | Take Home Message | Lecturer |
