# Supplementary material for: Conveying practical clinical skills with the help of teaching associates—a randomised trial with focus on the long term learning retention
Source: BMC Med Educ. 2017 Mar 28;17:65. doi: 10.1186/s12909-017-0892-5 (PMC5371235; doi:10.1186/s12909-017-0892-5)
Supplement: Supplementary file 2 — OSCE Checklist CMFS (Part A) - practical clinical skills. Figure S2. OSCE Checklist CMFS (Part B) - Global Rating Scale (GRS). (DOCX 19 kb) [file 12909_2017_892_MOESM2_ESM.docx]

Examination

Student information

**Examiner:**  ____________

**Part A**

| Examination | | Not attempted  0 | Attempted/Incomplete  1 | Correct/  Complete  2 |
| --- | --- | --- | --- | --- |
| Inspection (Describing the picture): monocle hematoma, anisocoria, nasal deviation, flattening/interruptions, swelling – if 3 and more pathologies are mentionend 2 points | |  |  |  |
| Eye | Pupils |  |  |  |
|  | Vision |  |  |  |
|  | Double vision |  |  |  |
| Nerves | Trigeminal |  |  |  |
|  | Facial |  |  |  |
| Skull | Skull cap |  |  |  |
| Midface | Orbital margin |  |  |  |
|  | Zygomatic bone |  |  |  |
|  | Le Fort I |  |  |  |
|  | Le Fort II |  |  |  |
|  | Le Fort III |  |  |  |
| Nose | Mobility |  |  |  |
|  | Inhibition of breathing |  |  |  |
|  | Septal hematoma |  |  |  |
| Lower jaw | Mandibular condyle |  |  |  |
|  | Collum/mandibular joint |  |  |  |
|  | Mandibular margin |  |  |  |
|  | Compression(chin) |  |  |  |
|  | Motility lower jaw |  |  |  |
| Intraoral | Oral inspection |  |  |  |
|  | Occlussion |  |  |  |
|  | Loose teeth |  |  |  |
|  | Opening of the mouth |  |  |  |

**Pay attention to Part B!**

**Part B**

|  | **1**  *Not attempted* | **2** | **3**  *Sufficient* | **4** | **5**  *Excellent* |
| --- | --- | --- | --- | --- | --- |
| Introduction | Does not state name |  | States name, forgets to inform about role/function and intention |  | States name, role/function and intention |
| Language/Description of the examination | Only uses technical terms without explaining them, interrupted speech, speaks very quietly |  | Tries to avoid technical terms, uses them often however. Interrupted speech once in a while. |  | The student uses a form of speech which is very comprehendible to the patient (does not use technical terms and if used explains them), uninterrupted speech with adequate speed and volume |
| Steps of the examination | Completely unstructured (e.g. changes frequently between inspection and functional tests), no structure recognisable |  | Basic structure in place, however with skips and insertions |  | Performs examination according to checklist |
| Appearance | Insecure and incompetent.  Scruffy personal appearance, bad hygiene |  | Nervous yet prepared |  | Secure, competent.  Neat personal appearance, good hygiene |
| Interaction with patient | Impolite, does not look at the patient, interrupts the patient frequently without moderating the conversation |  | Speaks to the examiner and patient alike, friendly most of the time.  However, interrupts the patient sometimes inadequately, sometimes looses eye contact |  | Friendly, politely engaged with the patient, keeps eye contact, does not interrupt patient and moderates the conversation, engages in the questions and worries of the patient. |
| Examination conditions | Does not care about examination conditions |  | Cares only moderately about examination conditions  (e.g. only closes window „pro forma“) |  | Creates adequate examination conditions (e.g. respects privacy, closes windows, checks the lighting, lets the patient remove his/her clothing adequately, optimal positioning,etc.) |
